# Incretin Hormones: The Link between Glycemic Index and Cardiometabolic Diseases

**DOI:** 10.3390/nu11081878

**Published:** 2019-08-13

**Authors:** Teresa Salvatore, Riccardo Nevola, Pia Clara Pafundi, Lucio Monaco, Carmen Ricozzi, Simona Imbriani, Luca Rinaldi, Ferdinando Carlo Sasso

**Affiliations:** 1 Unit of Internal Medicine, Department of Precision Medicine, University of Campania “Luigi Vanvitelli”, Piazza Miraglia, 2, 80138 Naples, Italy; 2 Unit of Internal Medicine, Department of Advanced Medical and Surgical Sciences, University of Campania “Luigi Vanvitelli”, Piazza Miraglia, 2, 80138 Naples, Italy

**Keywords:** glycemic index, incretin hormones, cardiometabolic diseases

## Abstract

This review aimed to describe the potential mechanisms by which incretin hormones could mediate the relationship between glycemic index and cardiometabolic diseases. A body of evidence from many studies suggests that low glycemic index (GI) diets reduces the risk for type 2 diabetes and coronary heart disease. In fact, despite the extensive literature on this topic, the mechanisms underlying unfavorable effects of high GI foods on health remain not well defined. The postprandial and hormonal milieu could play a key role in the relationship between GI and cardiovascular risk. Incretin hormones, glucagon-like peptide1 (GLP-1) and glucose-dependent insulinotropic polypeptide (GIP), are important regulators of postprandial homeostasis by amplifying insulin secretory responses. Response of GIP and GLP-1 to GI have been studied more in depth, also by several studies on isomaltulose, which have been taken as an ideal model to investigate the kinetics of incretin secretion in response to foods’ GI. In addition, extrapancreatic effects of these incretin hormones were also recently observed. Emerging from this have been exciting effects on several targets, such as body weight regulation, lipid metabolism, white adipose tissue, cardiovascular system, kidney, and liver, which may importantly affect the health status.

## 1. Introduction

High carbohydrates diets, especially from refined sources such as white rice and white bread, starches, and added sugars, are associated with an increased risk of cardiovascular events and all-cause mortality [1].

It has become clear that not all carbohydrates are the same and that post meal rise in glucose levels is mainly influenced by carbohydrates’ quality and other food-related compounds rather than by carbohydrate quantity per se.

The glycemic index (GI) is a measure of blood glucose increase elicited by foods, computed from the incremental area under the postprandial plasma glucose curve of a test food, expressed as percentage of that of an equal amount (typically 50 g, at times 25 g) of a reference carbohydrate (e.g., glucose or white bread) [2]. Even if its relevance still continues to be an object of debate, GI represents a property of the food itself, precisely defined by the International Organization for Standardization (ISO) method 26,642:2010 and a methodology sufficiently valid and reproducible for discriminating foods based on their glucose response [2].

Quality of carbohydrates as defined by GI can markedly affect the health status. In particular, consumption of high-GI foods has been described to increase the risk for noncommunicable diseases such as obesity, type 2 diabetes, and cardiovascular diseases [2,3]. Several prospective cohort studies suggest that low GI diets reduce the risk for type 2 diabetes and coronary heart disease [4,5,6].

Despite the extensive literature on this issue, the mechanisms underlying unfavorable effects of high GI foods on health still remain not well defined. The main role has been historically attributed to postprandial hyperglycemia, along with related hyperinsulinemia [7]. However, the postprandial metabolic and hormonal milieu is a very complex pathophysiological state, as many factors outside of blood glucose and insulin levels may be implicated. In this context, insulin secretory responses initiated by post-meal hyperglycaemia are notably amplified by incretin hormones, a phenomenon called “incretin effect” and attributed to the release of incretin hormones from specialized entero-endocrine cells elicited by absorption of oral glucose but not by intravenous glucose administration. Thus, incretin hormones, glucagon-like peptide-1 (GLP-1) and glucose-dependent insulinotropic polypeptide (GIP) may be important regulators of postprandial homeostasis [8].

The relationship between GI and healthy status is not clear, but several observations support a role for inflammatory as well as oxidative stress in foods with high GI. In particular, after a three-month reduction diet with low glycemic index, an increased level of IGF-I with cardioprotective effect was observed [9]. Moreover, a three-month intervention using a low glycemic index diet decreases inflammation by increasing the concentration of uric acid and the activity of glutathione peroxidase [10].

Since rates of glucose absorption closely related to GI of foods trigger a different pattern of incretin response, and a variety of pancreatic and extrapancreatic effects have been described for these gut-derived hormones, a role for GIP and GLP-1 in mediating GI effects on health status may be reasonably supposed. In this light, this review aimed to describe the potential mechanisms by which incretin hormones could mediate the relationship between glycemic index and cardiometabolic diseases.

## 2. Pancreatic Effects of Incretin Hormones

The majority of GIP producing K cells are found in the proximal small intestine and the duodenum, while GLP-1 producing L cells are much more dispersed, with a gradient from a low density in the duodenum to a higher density in the ileum, but also in the colon and rectum [11].

In fasting state, healthy human subjects have basal plasma concentrations for both incretins in the low picomolar range (10−12 mol L^−1^). These start to rise a few minutes after meal, reaching a peak after approximately 1 h, and returning to basal levels after several hours [11]. Nutrients stimulating incretin secretion are glucose and other carbohydrates (including sucrose and starch), triglycerides, and some aminoacids [12], whereas proteins are a comparatively weak stimulus.

Upon release, GIP and GLP-1 bind to specific G-protein coupled receptors present on ß-cells, where they exert additive effects on the stimulation of insulin secretion in a glucose-dependent manner [13]. Incretin hormones always require a permissive degree of hyperglycaemia to exert their insulinotropic action. Activation of protein kinase A elicited by the binding of incretins to their respective receptors cannot initiate the release of preformed insulin secretory granules from ß-cells, without closing of potassium channels, depolarization, and calcium ion influx, as determined by hyperglycemia. For the same reason, incretins cannot provoke episodes of hypoglycemia. For GLP-1, the absolute glycemic threshold below which insulin secretion cannot be stimulated, even at supra-physiological concentrations, has been identified as approximately 66 mg/dL [11]. This cut-off appears safe in particular in non-diabetic people, as these subjects generally show hypoglycemic symptoms for serum glycemic values below 50 mg/dL.

Incretin release is related to a meal by definition and depends on rates of nutrient entry into the small intestine to reach K and L cells [14]. Because of the more proximate location of GIP-producing K cells, the necessary gastric delivery, both in terms of food volume and glycemic content, is lower for GIP than GLP-1, even if studies in isolated perfused proximal small intestine suggest that both incretin responses to nutrients are elicited simultaneously [15].

Apart from insulin stimulation, GLP-1 reduces glucose concentrations by inhibiting α-cell glucagon secretion at all glucose levels and beyond the inhibition caused by blood glucose lowering alone [16]. GIP equally contributes with GLP-1 to the incretin effect, but unlike GLP-1 did not affect glucagon secretion.

In β-cells, GLP-1 not only enhances glucose-dependent insulin secretion and insulin synthesis, but, as described in animal studies, also stimulates β-cell proliferation and inhibits apoptosis, thus preserving β-cell function [17]. Whether this eventual regulation of β-cell mass in humans translates into a beneficial impact on diabetes progression still remains less elucidated.

Therefore, the pancreatic effects of GLP-1 and GIP are fundamentally similar, with the exception of the inhibitory effect on glucagon secretion performed only by GLP-1.

## 3. Relationship between Incretin Hormones and GI

### 3.1. Response of GIP and GLP-1 to Food Intake

Many studies have documented the response of incretin hormones to bread, reporting conflicting results. Fiber-enriched [18] and cereal-based [19] breads have been reported to reduce GIP secretion compared with white bread. A study [20] evaluating the five most common breads consumed in Spain, discovered that GIP release was higher after intake of wholemeal rather than white bread. Wheat bread consumption with different particle size elicited a GLP-1 response much lower after bread prepared with flour and 85% broken wheat kernels compared to a control bread made from wheat flour combined with wheat bran to obtain a similar fiber content [21].

Hartvigsen et al. [22] reported that only rye bread with kerornels may decrease GIP and GLP-1 postprandial secretion. Other authors have described no differential effect of white wheat or whole-grain breads on both incretins [23,24].

This wide variability of response probably depends on the fact that carbohydrates exert a different effect if ingested alone or into a food matrix such as bread. Many factors, among which manufacturing conditions, type of cereals, starch structure, bread particle size and inclusion of other ingredients, may affect the glycemic and incretin responses. In general, the glycemic response of a food is altered in the presence of other foods depending on the amount and source of carbohydrate and the amounts and types of fat and protein added. Moreover, other factors may influence entero-hormone release. Short chain fatty acids, produced during fermentation of unabsorbed carbohydrates or fiber by the intestinal microbiota, may stimulate GLP-1 secretion [25]. Bile acids are also able to potentiate GLP-1 release by activating their receptor TGR5 [26,27,28].

### 3.2. The Isomaltulose Model

Isomaltulose (6-O-D-glucopyranosyl-D-fructofuranose, Palatinose) is a disaccharide found in nature in honey and sugar cane, which has the same composition (one glucose molecule and one fructose molecule) and caloric value (4 kcal/g) as sucrose [29,30]. In industry, isomaltulose is manufactured using the enzyme glycosyltransferase, which converts the α-1,2 bond of sucrose into a α-1,6 bond [31].

Isomaltulose and sucrose are degraded respectively by isomaltase and sucrase, disaccharide-degrading enzymes located in small intestinal villi. In healthy subjects, isomaltulose is completely hydrolyzed at much lower rate than sucrose, thus determining slower rates of blood glucose and insulin increase as compared with sucrose [32,33]. The resulting monosaccharides (glucose and fructose) are completely absorbed by the small intestine [34,35] and do not reach the larger intestine, where alteration of microbioma might affect postprandial glucose responses [36]. Consequently, isomaltulose releases the same amount of energy as sucrose as confirmed in animal studies [32].

Based on these intrinsic characteristics, isomaltulose represents the ideal model to investigate the kinetics of incretin secretion in response to foods GI.

Studies on rats report that isomaltulose ingestion is characterized by reduced postprandial insulin and GIP release [37] and ileal administration of isomaltulose in anesthetized animals triggers a greater response of GIP than jejunal administration [38].

In the first human study [39] on the kinetics of incretin secretion elicited by isomaltulose, both plasma glucose and insulin levels resulted significantly lower, and total GIP secretion significantly and dramatically smaller, after isomaltulose than after sucrose loading. In contrast, GLP-1 levels observed at later time-points were significantly higher with isomaltulose than sucrose, as well as glucose and insulin levels at 120 min.

In a randomized, double-blind, crossover study on type 2 diabetic patients [40], postprandial glucose metabolism was characterized by using a combination of euglycemic-hyperinsulinemic clamp and labeled oral isomaltulose or sucrose load. Consistently with the previous report [39], absorption of isomaltulose was prolonged by on the 22-08-201950 min with respect to sucrose. Mean plasma concentrations of insulin, C-peptide, glucagon, and GIP were 10–23% lower. In contrast, GLP-1 was 64% higher after isomaltulose ingestion. Similarly, in another study [41] on type 2 diabetic participants, the incremental area under the curve of GIP was substantially reduced by 40% and that of GLP-1 remarkably and significantly 6.3-fold higher following isomaltulose than sucrose intake.

Keller et al. [42] reported that sugar sweetened beverages with isomaltulose (containing 50% fructose) led to significantly higher GLP-1 release compared to maltodextrin-sucrose intake (containing only 12.5% fructose), although GLP-1 response has been shown to be lower after pure fructose intake when compared with an equicaloric glucose load [43]. This result likely depends on the slower degradation of the α-1,6 glycosidic bond of isomaltulose and a higher proportion of glucose reaching the distal part of the ileum.

Therefore, in light of the above, low-GI carbohydrates are characterized by low postprandial endogenous GIP levels and increased GLP-1 concentrations. This most likely happens because slowly digested low-GI carbohydrates bypass the upper intestinal K-cells producing GIP and reach the most distant L-cells producing GLP-1.

### 3.3. Extrapancreatic Effects of Incretins

The GIP and GLP-1 receptors are widely expressed in multiple tissues and cell types [44] and a large body of evidence describes a plethora of pleiotropic activities for incretins outside of Langerhans islets. Most of these effects have been reported in animal experiments, and their relevance in humans have not always been ascertained [45].

### 3.4. GLP-1: The “Good” Incretin

#### 3.4.1. Body Weight Regulation

GLP-1 play a physiological control of body weight by reducing appetite and enhancing satiety. The exact mechanism of these effects is complex and not completely understood. Evidence has been accumulated to support roles for both central and peripheral GLP-1 in the regulation of energy balance.

Intravenous GLP-1 infusion decreases gastric emptying rate by means of afferent-mediated vagal central mechanisms [46]. The effect has been observed both in healthy human subjects [47] and in patients with type 2 diabetes [48] in a dose-dependent manner.

However, rather than primarily lowering gastrointestinal motor activity, GLP-1 mainly reduces appetite by affecting the brain regulating center’s function [49]. GLP-1 receptor is expressed in various brain areas consistent with regulation of appetite and satiety, and intracerebroventricularly administered GLP-1 strongly reduces short-term food intake in rats [50]. The afferent vagal nerve system is more likely a mediator of this central effect of GLP-1, as total subdiaphragmatic vagotomy attenuates the reduction in food intake induced by peripheral GLP-1 administration in rodents [51]. GLP-1 activity in the brain of peripheral GLP-1 could be mediated by afferent vagal nerve termini adjacent to L-cells and/or in the hepatoportal region [52].

In addition to effects on energy intake, GLP-1 may contribute to negative energy balance by increasing energy expenditure, as intracerebroventricular injection of GLP-1 increases thermogenesis from interscapular brown adipose tissue in mice [53].

#### 3.4.2. Lipid Metabolism

Fat ingestion is a physiological strong stimulator of GLP-1 release in humans and rodents [54]. On the other hand, GLP-1 infusion improves postprandial lipidemia [55], most likely as a result of delayed gastric emptying and insulin-mediated inhibition of lipolysis. In addition, a reduced Apolipoprotein B48 (Apo-B48) synthesis seems to be implicated. Apo-B48 is the primary protein component of the chylomicrons (CM)—it is specifically distributed in small intestine-derived CM. Its bloodstream concentration during fasting is usually quite low [56]. In rats, intravenously administered GLP-1 inhibits Apo-B48 production, resulting in decreased release of triglycerides into the circulation after lipid containing meals [57]. Exendin-4, a long-acting GLP-1 analogue, directly inhibits the synthesis of Apo-B48 from hamster enterocytes [58]. In this sense, the eventual postprandial hyperlipidemia, postprandial hyperglycemia, metabolic syndrome and myocardial infarction may be monitored and assessed by analyzing and monitoring Apo-B48 protein.

#### 3.4.3. White Adipose Tissue

Even though the presence of GLP-1 receptor (GLP-1R) in adipose tissue is controversial, GLP-1 exerts various effects at this level [59]. Both GLP-1 and exendin-4 stimulate insulin-mediated glucose uptake in isolated rat adipocytes [60].

GLP-1 robustly stimulates lipolysis in adipocytes isolated from abdominal fat of morbidly obese subjects [61], but not in subcutaneous abdominal fat of healthy volunteers [62]. Treatment with a GLP-1-producing adenovirus reduces fat mass, proinflammatory M1 macrophages and inflammatory cytokines in ob/ob mice, thus suggesting an anti-inflammatory action of GLP-1 in adipose tissue [63].

#### 3.4.4. Cardiovascular System

The GLP-1R has been found in various cardiovascular tissues and many studies indicate that GLP-1 have a host of protective effects at this level, independently of nutrient homeostasis [63,64].

In particular, endothelial dysfunction is believed to be an important link between the postprandial state, atherosclerosis and cardiovascular disease. Even if postprandial vasodilatation is mediated by insulin-induced release of nitric oxide [65], it has been demonstrated that GLP-1 per se has direct beneficial effects on endothelium-dependent vasodilatation, particularly in the postprandial state [66]. GLP-1 has been shown to increase NO availability in a wide range of vascular beds [67] and to inhibit endothelin-1 production [68]. In vitro, GLP-1 induces endothelium-dependent vasodilation in preconstricted pulmonary arteries [69] and inhibits TNF-alpha-mediated PAI-1 induction in vascular endothelial cells, improving cell dysfunction [70]. Administration of GLP-1 improves endothelial function in salt-sensitive hypertensive rats [71]. Of great relevance, pharmacological levels of GLP-1 improves endothelial function in healthy individuals [72] as well as in type 2 diabetic patients with stable coronary artery disease [73].

GLP-1 or GLP-1 receptor agonists have demonstrated multiple beneficial actions on the heart. In rats, GLP-1 protects myocardium from ischemia [74] and improves the cardiac function in animals with congestive heart failure [75]. In humans, GLP-1 attenuates ischemic left ventricular dysfunction during stress echocardiography in patients with coronary artery disease [76] and improves left ventricular function in some studies of heart failure subjects [77].

In patients with type 2 diabetes [78], long-term treatment with GLP-1 analogs reduces blood pressure, an effect well observed before significant weight loss and potentially mediated by GLP-1’s natriuretic effect and/or by improved endothelial function.

Notably, three CV outcome studies showed a significant reduction in a three-point MACE using GLP-1 analogs: liraglutide in LEADER, semaglutide in SUSTAIN-6, and recently dulaglutide in REWIND [79,80,81].

#### 3.4.5. Kidney

Intravenous GLP-1 infusion increases natriuresis in rats and humans [82], possibly via increased atrial natriuretic peptide secretion from the heart [83] or via increased expression of the Na+/H+ exchanger in renal tubules [84]. Since the GLP-1 receptor is expressed in the brush border microvilli of proximal renal tubules and glomerular endothelial cells, a direct modulation of Na+ handling by the kidney cannot be excluded.

### 3.5. GIP: The “Bad” Incretin

#### 3.5.1. White Adipose Tissue

Animal and human studies report a physiological role for GIP in the nutrient uptake into adipose tissues and, therefore, in the pathogenesis of obesity [85,86].

GIP may increase fat storage directly by binding to its receptors on adipocytes and indirectly by potentiating insulin secretion, which notoriously induce a switch from lipolysis to lipogenesis in the adipose tissue.

The fundamental support for the role of GIP in obesity comes from studies on GIP receptor knockout mice, which, unlike control animals, are protected from obesity and insulin resistance in response to high fat or high-GI diets [87]. Moreover, in a mice model of partial reduction of GIP secretion, high-fat diet alleviated obesity and lessened the degree of insulin resistance, accompanied by higher fat oxidation and energy expenditure [88].

There is also evidence that GIP induces both in mice and human fat cells expression and release of inflammatory cytokines with possible relapse on insulin resistance [89,90,91].

#### 3.5.2. Fatty Liver

Liraglutide, a GLP-1 analog, may improve liver fibrosis in non-alcoholic fatty liver disease [92]. On the contrary, increased postprandial release of GIP has be linked to unfavorable effects in this condition. In fact, in patients with NASH, GIP response correlated directly with hepatic steatosis, postprandial resistin, and free fatty acid (FFA) increase [93].

#### 3.5.3. Vascular Responses

There is strong evidence that GIP increases splanchnic perfusion after a meal to enhance blood supply to the gut and optimize nutrient delivery to the liver [94,95]. GIP may also be involved in non-splanchnic arterial regulation, likely by producing endothelin-1 and nitric oxide, as suggested by studies using cultured human endothelial cells [96].

In mouse arteries, GIP induces the expression of the proatherogenic cytokine osteopontin, a key player in the pathogenesis of vascular disease, through the local release of endothelin-1 and activation of CREB (a transcription factor participating in the regulation of osteopontin expression) [97].

Additional support for an unfavorable vascular role for GIP comes from a large-scale genome-wide association meta-analysis reporting the correlation of a variant in the GIP gene with myocardial infarction [98].

## 4. Conclusions

Plenty of evidence indicates that cardiometabolic health is affected by the quality of carbohydrate present in foods. As indicated by several studies exploring the incretin response to isomaltulose, low-GI carbohydrates are characterized by low postprandial endogenous GIP levels and increased GLP-1 concentrations. This probably is due to the different timing of stimulation of k-cells and L-cells, depending on the different GI of the carbohydrates.

Based on their opposite extrapancreatic effects, GIP and GLP-1 may behave as the “yin and yang” and could represent the causal link between GI and health status.

Currently, there is a lack of clinical trials investigating the impact of low-GI foods on body weight, glucose homeostasis and cardiovascular risk [3]. Moreover, the observed effects in intervention studies, when present, are generally of small magnitude.

However, a potential role in health outcome is suggested by the positive experience with alpha-glucosidase inhibitors [99,100], which convert meals into low-GI meals by shifting the sucrose absorption from the jejunal to ileal intestine, thus enhancing GLP-1 secretion [101,102].

In conclusion, it is reasonable to assume that incretin hormones play a crucial role in the attainment of a wide range of health benefits, as associated to the quality of ingested carbohydrates.

Currently, there are several physiopathological suggestions, but few strong clinical data, supporting this intriguing hypothesis, which, however, in light of recent important clinical evidence on the cardiometabolic protection effects of GLP1 receptor agonists, certainly deserve the development of ad hoc clinical trials.
